# All Roads Lead to Rome: Pathways to Engineering Disease Resistance in Plants

**DOI:** 10.1002/advs.202412223

**Published:** 2024-12-18

**Authors:** Aziz Ul Ikram, Muhammad Saad Shoaib Khan, Faisal Islam, Sulaiman Ahmed, Tengfang Ling, Feng Feng, Zongtao Sun, Huan Chen, Jian Chen

**Affiliations:** ^1^ International Genome Center Jiangsu University Zhenjiang 212013 China; ^2^ Plant Systems Engineering Research Center Korea Research Institute of Bioscience and Biotechnology (KRIBB) Yuseong‐gu Daejeon 34141 Republic of Korea; ^3^ Department of Biochemistry and Molecular Biology Oklahoma State University Stillwater OK 74078 USA; ^4^ State Key Laboratory for Managing Biotic and Chemical Threats to the Quality and Safety of Agro‐products, Key Laboratory of Biotechnology in Plant Protection of Ministry of Agriculture and Zhejiang Province, Institute of Plant Virology Ningbo University Ningbo 315211 China; ^5^ Joint Center for Single Cell Biology, School of Agriculture and Biology Shanghai Jiao Tong University 800 Dongchuan Road Shanghai 200240 China

**Keywords:** Disease resistance, *E* genes, Genetic engineering, NLRs, Plant immunity, PRRs, *S* genes, SAR

## Abstract

Unlike animals, plants are unable to move and lack specialized immune cells and circulating antibodies. As a result, they are always threatened by a large number of microbial pathogens and harmful pests that can significantly reduce crop yield worldwide. Therefore, the development of new strategies to control them is essential to mitigate the increasing risk of crops lost to plant diseases. Recent developments in genetic engineering, including efficient gene manipulation and transformation methods, gene editing and synthetic biology, coupled with the understanding of microbial pathogenicity and plant immunity, both at molecular and genomic levels, have enhanced the capabilities to develop disease resistance in plants. This review comprehensively explains the fundamental mechanisms underlying the tug‐of‐war between pathogens and hosts, and provides a detailed overview of different strategies for developing disease resistance in plants. Additionally, it provides a summary of the potential genes that can be employed in resistance breeding for key crops to combat a wide range of potential pathogens and pests, including fungi, oomycetes, bacteria, viruses, nematodes, and insects. Furthermore, this review addresses the limitations associated with these strategies and their possible solutions. Finally, it discusses the future perspectives for producing plants with durable and broad‐spectrum disease resistance.

## Graphical abstract

Crops lost to diseases necessitate a deeper understanding of plant immunity and the development of disease resistance. Here, the arms‐race between plants and pathogens is briefly described, and a comprehensive overview of the current and emerging strategies for engineering disease resistance in plants is provided. An essential comparison among different strategies to aid plant scientists in practical application or future research is also provided.

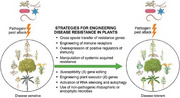

## 1 Introduction

Both plants and animals, despite the fundamental differences between them, are exposed to a multitude of pathogens that cause diseases in them. Plants, being sessile in nature, are particularly vulnerable to a plethora of disease‐causing pathogens and harmful pests, including filamentous pathogens (fungi and oomycetes), bacteria, viruses, nematodes, and insects. Unlike animals, plants lack circulatory system, and their cells are surrounded by a rigid cell wall. Due to these evolutionary constraints, plants have evolved a cell‐autonomous immune system, that consists of immune recognition by immune receptors present at the cell surface and inside the cell, followed by a complex signaling network leading to defense response. Additionally, plants can fend off pathogens by RNA interference (RNAi) and autophagy, as well as through systematic acquired resistance (SAR) in the uninfected parts. In turn, pathogens have evolved various strategies that help them avoid plant defense mechanisms or by subverting them to promote infection, resulting in huge yield losses annually.^[^ ^1^ ^]^ A recent study found that the global crop losses caused by pathogens and pests range from 10.1% to 28.1% in wheat (*Triticum aestivum*), 19.5% to 41.1% in maize (*Zea mays*), 24.6% to 40.9% in rice (*Oryza sativa*), 11.0% to 32.4% in soybean (*Glycine max*) and 8.1% to 21.0% in potato (*Solanum tuberosum*).^[^ ^2^ ^]^ Until now, agrochemicals have been widely used for directly eliminating pathogens and pests. However, they pose significant harm to both humans and the environment.^[^ ^3^ ^]^ Developing new cultivars with stable and durable resistance to pathogens is the most economical as well as eco‐friendly way to deal with this problem.

The diverse aspects of pathogen/pest and plant interactions provide opportunities for engineering disease resistance in plants. Our capacity for plant disease management is enhanced further by the latest technologies of gene manipulation, including genome editing and synthetic biology. Unlike traditional breeding methods, using modern transgenic approaches, we can precisely transfer single resistance gene or combine multiple resistance genes in a single plant in short time and avoid the problem of linkage drag. Domains swapping in pattern recognition receptors (PRRs) and ability to modify or extend the effector recognition specificity of NOD‐like receptors (NLRs) further enhance our ability to engineer disease resistant plants.^[^ ^4^ ^]^ Enhancing the activity of the positive regulators of plant immunity such as plant hormones, transcriptional regulators, mitogen‐activated protein kinase (MAPK/MPK) cascades, as well as inducing SAR can provide broad‐spectrum resistance against pathogens.^[^ ^5^ ^]^ Disrupting the function of *susceptibility (S)* genes through gene editing can provide disease resistance in non‐transgenic system.^[^ ^6^ ^]^ Activation of RNAi can provide a powerful strategy to control many plant viruses by targeting viral RNA for degradation.^[^ ^1^ ^]^ Autophagy in plants can directly degrade viral components, making it a promising strategy to control plant pathogens particularly viruses.^[^ ^1^ ^]^ While these technologies offer great potential, it is important to address potential limitations such as autoimmunity associated with NLRs engineering and positive regulators of immunity, and fitness cost associated with *S* gene editing. Beside genetic approaches, the use of beneficial microbes and organic methods are becoming popular among farmers as eco‐friendly and sustainable strategies for plant disease management.^[^ ^7^ ^]^

In this review article, we provide a basic summary of the fundamental mechanisms of the arms‐race between pathogens/pests and host plants, and provide a detailed overview of current and emerging strategies for engineering disease resistance in plants. We expect this review to serve as a helpful guide for plant scientists to directly apply current advances in the knowledge of plant‐pathogen interactions for breeding purposes or to conduct similar research in other crops.

## 2 Plants versus Pathogens: The Tug‐of‐War for Survival Between Plants and Pathogens

Plants rely on a multi‐layered defense system to protect themselves from pathogens. At the surface of the cell, plants have PRRs that recognize pathogen/microbe‐associated molecular patterns (PAMPs/MAMPs), such as bacterial flagellin, peptidoglycans, and fungal cell wall chitin or damage‐associated molecular patterns (DAMPs), such as plant cell wall fragments.^[^ ^8^ ^]^ The immunity triggered by the recognition of PAMP/DAMP by PRRs is called pattern‐triggered immunity (PTI) that involves calcium (Ca^2+^) influx, generation of reactive oxygen species (ROS), secretion of antimicrobial substances and hydrolytic enzymes (e.g., glucanases and chitinases) that target cell walls of the pathogens, induction of callose deposition and transcriptional changes.^[^ ^9^ ^]^ Host non‐​adapted pathogens that are incapable of subverting the host's immune system are often unable to evade PTI. By using various strategies, including degrading, altering, sequestering, or preventing the release of PAMPs, host‐adopted pathogens prevent PRR recognition and activation to evade PTI and successfully infect their hosts.^[^ ^1^ ^]^ Additionally, they secrete so‐called effectors proteins into the intercellular spaces (apoplast) or inside the host cells to prevent recognition by PRRs and compromise downstream signaling of PTI, causing susceptibility.^[^ ^1^, ^10^ ^]^ Apoplastic effectors interfere with the binding of PAMPs or DAMPs to PRRs. The microbial effectors released into the plant cells can be recognized either directly by the plant NLRs or indirectly through their modifications of host proteins (guardees/decoys), which are also monitored by NLRs. NLRs, which are primary plant resistance (R) proteins, form the second layer of plant immunity called effector‐triggered immunity (ETI), which often involves a local cell death also known as hypersensitive response (HR).^[^ ^8^, ^11^ ^]^ In plants, in addition to intracellular recognition, cell wall localized NLR Rsc4‐3 is reported to trigger resistance against *soybean mosaic virus* (SMV) by recognizing SMV cylindrical inclusion (CI) effector in the apoplast.^[^ ^12^ ^]^ Despite the presence of a stringent NLRs‐based recognition in plants, pathogens regain pathogenicity and impair ETI/PTI signaling and response, by their ability to undergo rapid evolution in the effectors coding avirulence (AVR) genes, modulation of guardees/decoys, or acquisition of epistatic effectors.^[^ ^1^ ^]^

In addition to PTI and ETI, which provide a primary and local response to inhibit pathogen infection, a secondary defense response, called systemic acquired resistance (SAR), in the uninfected parts of the plant can also be triggered that provides broad spectrum resistance to a variety of pathogens.^[^ ^1^ ^]^ Two parallel and interconnected pathways, one triggered by plant defense hormone salicylic acid (SA) and the other triggered by the non‐proteinogenic amino acid pipecolic acid (Pip) or its presumed bio‐active derivative *N*‐hydroxy‐Pip (NHP), activate SAR.^[^ ^13^ ^]^ SAR is often associated with the induction of a group of *pathogenesis‐related* (*PR*) genes that determine resistance.^[^ ^1^ ^]^ Pathogens interfere with SAR in various ways including using effectors that inhibit the biosynthesis of SA or cause degradation of SA.^[^ ^14^ ^]^ Effectors also target *non‐expressor of pathogenesis‐related genes 1* (*NPR1*), which is a key regulator of the SA pathway and is required for the activation and expression of *PR* genes.^[^ ^15^ ^]^

Plants can also fend off pathogens by RNAi and autophagy. RNAi is the major defense mechanism in plants against viruses. Plants with defective RNAi pathway are more susceptible to viral infections. In turn, viruses encode suppressor proteins which can block the host silencing pathway thereby promoting viral replication.^[^ ^1^ ^]^ Autophagy, a conserved intracellular pathway through which unwanted cellular material is degraded, also modulates the plant response to pathogen infection particularly viral infection.^[^ ^16^ ^]^ Autophagy can directly degrade viral components, and restrict viral replication in plants. Plant viruses can counter host defenses by targeting host defense factors for the degradation.^[^ ^17^ ^]^

**Figure 1:**
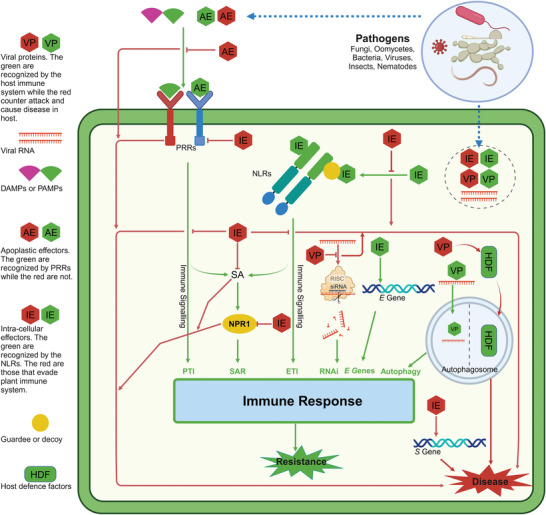
The basic mechanism of pathogenesis. The red arrows show pathways to disease while the green arrows show pathways to resistance. The pathogen‐associated molecular patterns (PAMPs) and damage‐associated molecular patterns (DAMPs) from the pathogens or host plants are detected by pattern recognition receptors (PRRs) that induce pattern‐triggered immunity (PTI) in plants. Pathogens, however, release apoplastic effectors and intracellular effectors. The apoplastic effectors interfere with PAMPs/DAMPs‐triggered immunity, causing disease. The intracellular effectors are detected by intracellular or cell wall localized receptors called NOD‐like receptors (NLRs), that trigger effector‐triggered immunity (ETI). However, pathogens evolve effectors that evade ETI and suppress PTI/ETI by interfering with recognition, signaling, and defense response, causing disease. PTI and ETI trigger a systematic response called systemic acquired resistance (SAR) in non‐infected parts of the plants. Salicylic acid (SA) and non‐expressor of pathogenesis‐related genes 1 (NPR1) are key components of SAR. Pathogen effectors interfere with SAR by inhibiting SA biosynthesis, causing SA degradation, or targeting NPR1. Plants can also fend off pathogens, particularly viruses by RNA interference (RNAi) and autophagy. In turn, plant viruses have evolved proteins that can block the host silencing pathway or exploiting host autophagic pathway by targeting host defense factors for the degradation. Some pathogens also manipulate host genes called *susceptibility* (*S*) genes for a successful infection. To counteract *S* genes, plants have evolved *executer* (*E*) genes, that can specifically trap certain transcription activator‐like effectors (TALEs) and trigger strong hypersensitive response (HR).

Some pathogens also manipulate host genes that facilitate infection and support compatibility. The so‐called *S* genes of the host are often targeted by effectors to facilitate infection.^[^ ^6^ ^]^ Effectors targeting *S* genes help the pathogens enter the host, disable the host defense systems, or increase nutrient availability for pathogens.^[^ ^6^ ^]^ To counteract *S* genes, specifically those induced by transcription activator‐like effectors (TALEs), some plants have evolved *executor* (*E*) genes, which can trap certain TALEs that trigger strong HR responses^[^ ^18^ ^]^ (**Figure** **1**).

Besides defense within plants, there exists defense between plants. For instance, plants under pathogen/pest attack also secrete certain compounds such as root exudates or volatile organic compounds (VOCs) that can repel the attacker, attract beneficial microbiota to their rhizosphere or attract predatory arthropods in case of herbivore attack (Cry‐for‐help). The neighboring plants can also perceive such compounds and become alert to the risk of attack.^[^ ^19^ ^]^ Meanwhile, herbivorous have adopted to withstand plant defenses, and in some cases, they sequester and reuse these compounds in their defense.^[^ ^20^ ^]^ This suggests the continuous evolution of defense strategies between plants and pests/ pathogens.

## 3 Resistance Engineering in Plants

### 3.1 Cross‐Species Transfer of Resistance Genes

#### 3.1.1 Transfer of Single‐Resistance Gene

Resistance can be engineered by the transfer of single gene coding for cell‐surface immune receptors/PRRs or intra‐cellular immune receptors/NLRs (**Table** **1**). Plants' PRRs are popular for transferring disease resistance because of their ability to recognize common signatures of pathogens/pests and trigger conserved signaling pathways. For example, the Agrobacterium (Agrobacterium tumefaciens)‐mediated transfer of receptor‐like gene, *Vf*, for resistance to apple scab caused by *Venturia inaequalis*, has been reported in several studies.^[^ ^21^ ^]^ Transfer of Cf‐9, another resistance gene, from tomato to potato and tobacco plants resulted in an increased HR response to Cladosporium fulvum expressing Avr 9.^[^ ^22^ ^]^ Transgenic banana, sweet orange, or tomato plants expressing rice PRR, *Xa21*, exhibited resistance against *Xanthomonas* sp.^[^ ^23^ ^]^ Similarly, the EFR receptor, naturally limited to *Brassicaceae*, was transferred from Arabidopsis (*Arabidopsis thaliana*) to tomato and rice to generate novel resistance to bacteria.^[^ ^24^ ^]^ Likewise, an allelic FLS2 receptor, transferred from wild grape to tobacco resulted in enhanced resistance to the crown‐gall disease caused by Agrobacterium.^[^ ^25^ ^]^

**Table 1:** Cross‐species transfer of resistance genes to develop resistance.

Gene name	Source plant species	Target plant species	Pathogen name	Reference
PRRs				
*Vf*	*Malus floribunda*	*Malus Domestica*	*Venturia inaequalis*	[21]
Cf‐9	*Solanum lycopersicum*	*Solanum tuberosum*, *Nicotiana benthamiana*	Cladosporium fulvum	[22]
*Xa21*	*Oryza longistaminata*	Banana, *Citrus sinensis*, *Solanum lycopersicum*	*Xanthomonas* sp.	[23]
*EFR*	*Arabidopsis thaliana*	*Solanum lycopersicum*	*Ralstonia solanacearum, Xanthomonas perforans*	[24]
*EFR*	*Arabidopsis thaliana*	*Triticum aestivum*	*Pseudomonas syringae* pv. *oryzae*	[53]
*EFR*	*Arabidopsis thaliana*	*Oryza sativa*	Escherichia coli, Xanthomonas oryzae p*v*. oryzae	[54]
*FLS2*	*Vitis riparia*	*Nicotiana benthamiana*	*Agrobacterium tumefaciens*	[25]
NLRs				
Bs2	*Capsicum annuum*	*Solanum lycopersicum*	Xanthomonas species	[27]
Rpi‐vnt1.1	*Solanum venturii*	*Solanum tuberosum*	Phytophthora infestans	[28]
Rxo1	*Zea mays*	*Oryza sativa*	*Xanthomonas oryzae* pv. *oryzicola*	[29]
*Rpi‐blb1*	*Solanum bulbocastanum*	*Solanum tuberosum*, *Solanum lycopersicum*	Phytophthora infestans	[30]
*Mi‐1.2*	*Solanum lycopersicum*	*Solanum melongena*	*Meloidogyne javanica* (nematode)	[31]
*CcRpp1*	Pigeonpea (*Cajanus cajan*)	*Glycine max*	*Phakopsora pachyrhizi*	[32]
*RPS4* and *RRS1*	*Arabidopsis thaliana*	*Brassica rapa, Brassica napus, Nicotiana benthamiana, Solanum lycopersicum*	*Colletotrichum higginsianum*, Pseudomonas syringae pv. Tomato DC3000, Ralstonia solanacearum	[55]
Roq1	*Nicotiana benthamiana*	*Solanum lycopersicum*	*Xanthomonas species*, *Pseudomonas syringae* and *Ralstonia species*	[33]
Bs2	*Capsicum chacoense*	*Citrus sinensis*	*Xanthomonas citri* subsp. *citri*	[35]
*MLA1*	*Hordeum vulgare*	*Arabidopsis thaliana*	Blumeria graminis f. sp. hordei	[36]
Stacking (*Sr22, Sr35, Sr45, Sr50, Sr55*)	*Triticum boeoticum, Triticum monococcum, Aegilops tauschii, Secale cereale, Triticum aestivum*	*Triticum aestivum*	*Puccinia graminis* f. sp. *tritici*	[49]
Stacking (*Sr22*, *Sr33*, *Sr35, Sr45*)	*Triticum boeoticum, Triticum monococcum, Aegilops tauschii*	*Hordeum vulgare*	*Puccinia graminis* f. sp. *tritici*	[50]
Stacking (*RB*,* Rpi‐blb2, Rpi‐vnt1.1*)	Solanum bulbocastanum and Solanum venturii	*Solanum tuberosum*	Phytophthora infestans	[51]
Stacking (*Pm3a Pm3b* or *Pm3b Pm3f*)	*Triticum aestivum*	*Triticum aestivum*	Blumeria graminis f. sp. tritici	[52]
Non‐immune receptor genes				
RPW8	*Arabidopsis thaliana*	*Oryza sativa*	Magnaporthe oryzae, Xanthomonas oryzae pv. oryzae	[40]
Lr34res	*Triticum aestivum*	*Hordeum vulgare*	*Puccinia hordei*, *Blumeria graminis* f.sp. *hordei (Bgh)*	[43]
Lr34res	*Triticum aestivum*	Sorghum bicolor	Puccinia purpurea, Colletotrichum sublineolum	[43]
Lr34res	*Triticum aestivum*	*Zea mays*	Exserohilum turcicum, Puccinia sorghi	[43]

Being the primary detectors of intracellular effectors, NLRs also offer opportunities to confer resistance when transferred among closely related species.^[^ ^26^ ^]^ Transfer of pepper *Bs2* gene to tomato conferred resistance to bacterial spot disease caused by Xanthomonas strains harboring avrBs2.^[^ ^27^ ^]^ Transgenic potato plants expressing the wild potato *R* gene *RB* or *Rpi‐vnt1.1*, showed resistance to potato late blight caused by *Phytophthora* infestans (*P. infestans*).^[^ ^28^ ^]^ In addition, introducing maize *Rxo1* in rice provided resistance against *Xanthomonas* oryzae pv. oryzicola (*Xoc*) which causes bacterial streak.^[^ ^29^ ^]^ The transfer of *Rpi‐blb1*, an ancient gene derived from the wild potato (*Solanum* *bulbocastanum*), provided broad‐spectrum resistance to *P. infestans*.^[^ ^30^ ^]^ Tomato *Mi‐1.2* gene provided resistance to root‐knot nematode, *Meloidogyne javanica*, in genetically engineered eggplants.^[^ ^31^ ^]^ Transgenic soybean lines expressing pigeon pea (*Cajanus cajan*) gene, *Cajanus cajan Resistance against Phakopsora pachyrhizi 1, CcRpp1*, conferred resistance to *Phakopsora pachyrhizi*.^[^ ^32^ ^]^ Transgenically expressing *Roq1* from tobacco in tomato showed bacterial wilt resistance.^[^ ^33^ ^]^

Although NLR transfer among closely related species is common, limited success is reported across broader phylogenetic distances. The pepper *Bs2* gene confers resistance against *Xanthomonas* *campestris* pv. *Vesicatoria* (*Xcv*) strains containing avrBs2. The *avrBs2* gene *from Xanthomonas* is highly conserved sharing 96% homology with the *avrBs2* from *Xanthomonas citri* subsp. *Citri* (*Xcc*), the causative agent of citrus canker.^[^ ^34^ ^]^ Transgenic sweet orange (*Citrus sinensis*) lines expressing the pepper *Bs2* gene conferred resistance to citrus canker.^[^ ^35^ ^]^ Similarly, Arabidopsis expressing barley (*Hordeum vulgare*) NLR, *Mildew Resistance Locus A* (*MLA1*) showed immunity against barley powdery mildew fungus expressing the corresponding effector *AVRA1*.^[^ ^36^ ^]^ The interactions among NLRs and downstream signaling components, probably limit the function of NLRs across broader phylogenetic distances. This problem can be solved to some extent by expressing downstream partners together with NLRs in heterologous systems.^[^ ^37^ ^]^

In addition to developing resistance through cross‐species transfer of immune receptors, there are several other dominant genes that have been identified and utilized for engineering disease resistance in plants. For example, *RESISTANCE TO POWDERY MILDEW 8* (*RPW8*), initially identified in Arabidopsis, to confer resistance to multiple isolates of powdery mildew,^[^ ^38^ ^]^ is a defense regulator involved in NLR activation and boosting *WRKYs* expression.^[^ ^39^ ^]^ Ectopic expression of *RPW8.1* in Arabidopsis provides resistance to virulent powdery mildew, oomycete, and bacterial pathogens.^[^ ^40^ ^]^ Heterologous expression of *RPW8.1* in rice led to enhanced resistance to fungal blast and bacterial blight.^[^ ^40^ ^]^ Other examples of non‐immune receptor resistance genes are those coding for ATP‐binding cassette transporters (ABC transporters) that mediate the transport of secondary metabolites, stress hormones, xenobiotics, or function as stress response gene regulators.^[^ ^41^ ^]^ The *Lr34res* allele of wheat ABC transporter Lr34 is associated with ABA transport and provides durable resistance to powdery mildew as well as rust diseases.^[^ ^42^ ^]^ The *Lr34res*, besides being one of the durable sources of quantitative fungal resistance in wheat, provide disease resistance when expressed in barley, maize, and sorghum (*Sorghum bicolor*).^[^ ^43^ ^]^ Thus, *RPW8.1* and *Lr34res* can be used for engineering disease resistance in crops.

Transfer of resistance genes often involve regeneration from callus. However, it is technically challenging in many species, particularly in monocots.^[^ ^44^ ^]^ Therefore, efficient transformation systems should be developed for species recalcitrant to transformation. More than 60 immunity‐related PRRs with known ligands and more than 140 NLRs with known recognized effectors have been identified in plants.^[^ ^45^ ^]^ Discovery of more PRRs/NLRs would provide additional resources to engineer crops resistance against multiple pathogens. The restricted taxonomic functionality poses a significant challenge to transferring *NLR* genes from model species to crops, however a deeper understanding of *NLR* gene signaling can find a solution to this problem.^[^ ^46^ ^]^

#### 3.1.2 Stacking Multiple Resistance Genes

**Figure 2:**
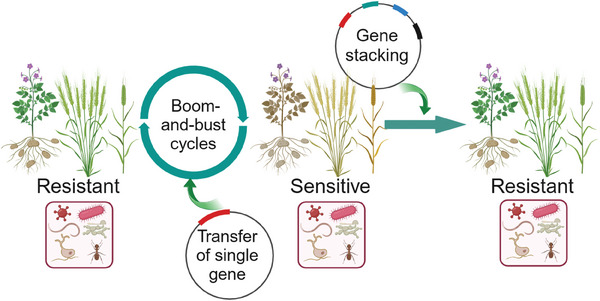
Boom‐and‐bust cycles associated with single gene transfer and generation of stable resistance through gene stacking. For example, single gene transfer to develop resistance to wheat stem rust of barley and wheat or late blight of potato is overcome due to the rapid evolution of pathogen (left panel). However, gene stacking by introducing a transgene cassette of multiple resistance genes as a single locus produced durable resistance (right panel).

Sometimes the effect of single gene transfer for resistance is not durable because of the low recognition range of pathogens and rapid evolution of pathogen effectors. Transferring multiple resistance genes or several alleles of the same resistance gene, called gene stacking also known as gene pyramiding, often overcomes this problem (**Figure** **2**). Pyramiding, potentially, prevents or delays the so‐called boom‐and‐bust cycles commonly associated with the transfer of a single resistance gene.^[^ ^47^ ^]^ For example, *Puccinia graminis* f. sp. *tritici* (*Pgt*), which causes wheat stem rust, a destructive fungal disease, significantly reduces plant growth and yield of wheat and barley.^[^ ^48^ ^]^ Breeding wheat with durable *Pgt* resistance is challenging due to the fast evolution of pathogen virulence. Bread wheat lines generated by transferring a transgene cassette consisting of five resistance genes as single locus, conferred resistance to *Pgt* isolates from all over the world.^[^ ^49^ ^]^ Transgenic lines generated by stacking wheat *Sr22*, *Sr33*, *Sr35*, and *Sr45* genes in barley showed strong resistance to *Pgt*.^[^ ^50^ ^]^ Potato late blight caused by *P. infestans* is one of the most devastating diseases that causes yield losses of ≈15%‐30% annually in sub‐Saharan Africa. Resistance developed through the transfer of single *R* genes from the wild potato species, *Solanum demissum*, has been rapidly overcome by the continuous evolution of pathogen strains. Potato lines that showed complete and durable resistance to local blight races in the field, were generated through the transfer of a gene stack consisting of three *R* genes from wild relatives (*Rpi‐vnt1.1* from *Solanum venturi* and *RB*, *Rpi‐blb2* from *Solanum bulbocastanum*).^[^ ^51^ ^]^ In another example, pyramiding *Pm3* alleles, *Pm3a/Pm3b* or *Pm3b/Pm3f*, in wheat, provided complete resistance to powdery mildew in field trials of two years.^[^ ^52^ ^]^ A potential disadvantage of gene stacking is the exertion of strong selection pressure on microbial pathogens that may lead to the evolution of “super pathogens” and can result in uncontrollable disease outbreaks.

### 3.2 Engineering Immune Receptors

#### 3.2.1 Domain Swapping and Chimeric PRRs

PRRs engineering offers an attractive strategy to develop durable and broad‐spectrum disease resistance in plants. There are two types of plant PRRs, receptor‐like proteins (RLPs) and receptor‐like protein kinases (RLKs). RLPs contain an extracellular ligand‐binding domain and a transmembrane domain, while RLKs, in addition to a ligand‐binding extracellular domain and a transmembrane domain, also contain a signal‐transmitting intracellular kinase domain.^[^ ^56^ ^]^ By swapping and recombining different domains of PRRs, the recognition specificity and strength of PRRs can be boosted in the domains donated plants (**Table** **2**). For instance, transgenic rice expressing chimeric receptors combining the rice chitin‐binding protein Chitin Elicitor‐Binding Protein (CEBiP) with the intracellular protein kinase domain of the rice receptor‐like kinase *Xa21* or *Pi‐d2* exhibited resistance to blast fungus, *Magnaporthe oryzae* (*M. oryza*e).^[^ ^4^, ^57^ ^]^ In another example, a functional chimeric receptor in both Arabidopsis and rice was obtained by fusing the ectodomain of the Arabidopsis EF‐Tu receptor (EFR) to the intracellular domain of rice Xa21 receptor.^[^ ^54^, ^58^ ^]^ Conversely, fusing the ectodomain of rice XA21 to the cytoplasmic domain of Arabidopsis EFR in transgenic rice provided resistance to *Xanthomonas oryzae* pv*. oryzae* (*Xoo*).^[^ ^59^ ^]^

**Table 2:** Engineered immune receptors to develop resistance in plants.

Gene (s)	Type of engineering	Plant Species	Pathogen	Reference
PRRs				
*CEBiP* and *Xa21*	Chimera	*Oryza sativa*	*Magnaporthe oryzae*	[4, 57]
*EFR* and *Xa21*	Chimera	*Arabidopsis thaliana*, *Oryza sativa*	Xanthomonas oryzae pv. oryzae	[54, 59]
*ReMAX* and *Eix2*	Chimera	*Nicotiana benthamiana*	*Xanthomonads*	[62]
*EFR* and *Cf‐9*	Chimera	*Nicotiana benthamiana*	Pseudomonas amygdali pv. Tabaci (Pta) 11 528 and Pseudomonas syringae pv. Tomato DC3000	[63]
NLRs				
*Rx*	Mutations in the LRR domain	*Nicotiana benthamiana*	*Potato virus X* (PVX) strains	[67]
Sr35	Mutations in the LRR domain	*Triticum aestivum* leaf protoplasts	*Puccinia graminis* f. sp. *tritici* that express AvrSr35	[68]
*Sr33*	Targeted engineering of LRR domain	*Triticum aestivum* leaf protoplasts	*Puccinia graminis* f. sp. *tritici* effector AvrSr50	[87]
*Pikp*	HMA domain engineering	*Nicotiana benthamiana*	AVR‐Pik variants	[72]
*RGA5*	HMA domain engineering	*Oryza sativa*	*Magnaporthe oryzae* Avrs‐ and ToxB‐like effector AvrPib	[88]
*RGA5*	HMA domain engineering	*Oryza sativa*	Magnaporthe oryzae strains expressing AVR‐PikD	[89]
*Pikp‐1, HIPP19*	HMA domain exchanged	*Oryza sativa*	*Magnaporthe oryzae* isolates carrying AVR‐PikC or AVR‐PikF	[75]
*Pik‐1*	HMA domain replaced with nanobodies	*Nicotiana benthamiana*	Fluorescent proteins (FPs)	[76]
*PBS1*	Decoy engineering	*Arabidopsis thaliana*, *Nicotiana benthamiana*	*Pseudomonas syringae* effector, AvrRpt2	[77]
*PBS1*	Decoy engineering	*Glycine max* protoplasts	Soybean mosaic virus (SMV)	[78]
*PBS1*	Decoy engineering	*Arabidopsis thaliana* and *Glycine max*	Potyvirus	[78]
*PBS1*	Decoy engineering	*Solanum tuberosum*	*Potato virus Y* (PVY)	[79]
*RRS1‐R/RPS4*	Synthetic NLR circuit	*Nicotiana benthamiana*	Phytoplasma effector SAP05	[80]
*Rx*	Random mutagenesis in the NB‐ARC domain	*Nicotiana benthamiana*	*Poplar mosaic virus* (PoMV)	[83]
*Sw5b*	Artificial evolution involving a sensitizing mutation in the LRR domain	*Solanum lycopersicum*	*Tomato spotted wilt virus* (TSWV)	[84]
*NRC2*	Targeted engineering of NRC2‐SS15 binding interface	*Nicotiana benthamiana*	*Globodera rostochiensis*	[86]

The problems of interfamily transfer of PRRs can also be potentially solved by domain swapping of PRRs. For example, a proteinaceous PAMP, eMAX from *xanthomonads* is detected by RLP1/ReMAX in Arabidopsis.^[^ ^60^ ^]^ ReMAX/RLP1 shows structural similarity to RLP Eix2 from tomato, which detects fungal xylanase as PAMP.^61^ A chimeric receptor by fusing the ectodomain of ReMAX with the C‐terminal part of Eix2, made interfamily transfer of eMax perception possible in tobacco that previously did not respond to eMax.^[^ ^62^ ^]^ Similarly, the transient and stable expression of chimeric EFR‐Cf‐9 receptor, consisting of the ectodomain of the Arabidopsis EFR receptor and the endodomain of the tomato Cf‐9 protein triggered a robust HR response to elf18 treatment in tobacco.^[^ ^63^ ^]^ These studies suggest that domain swapping to make chimeric immune receptors is a promising strategy to enhance disease resistance in plants.

#### 3.2.2 Engineering NLRs

**Figure 3:**
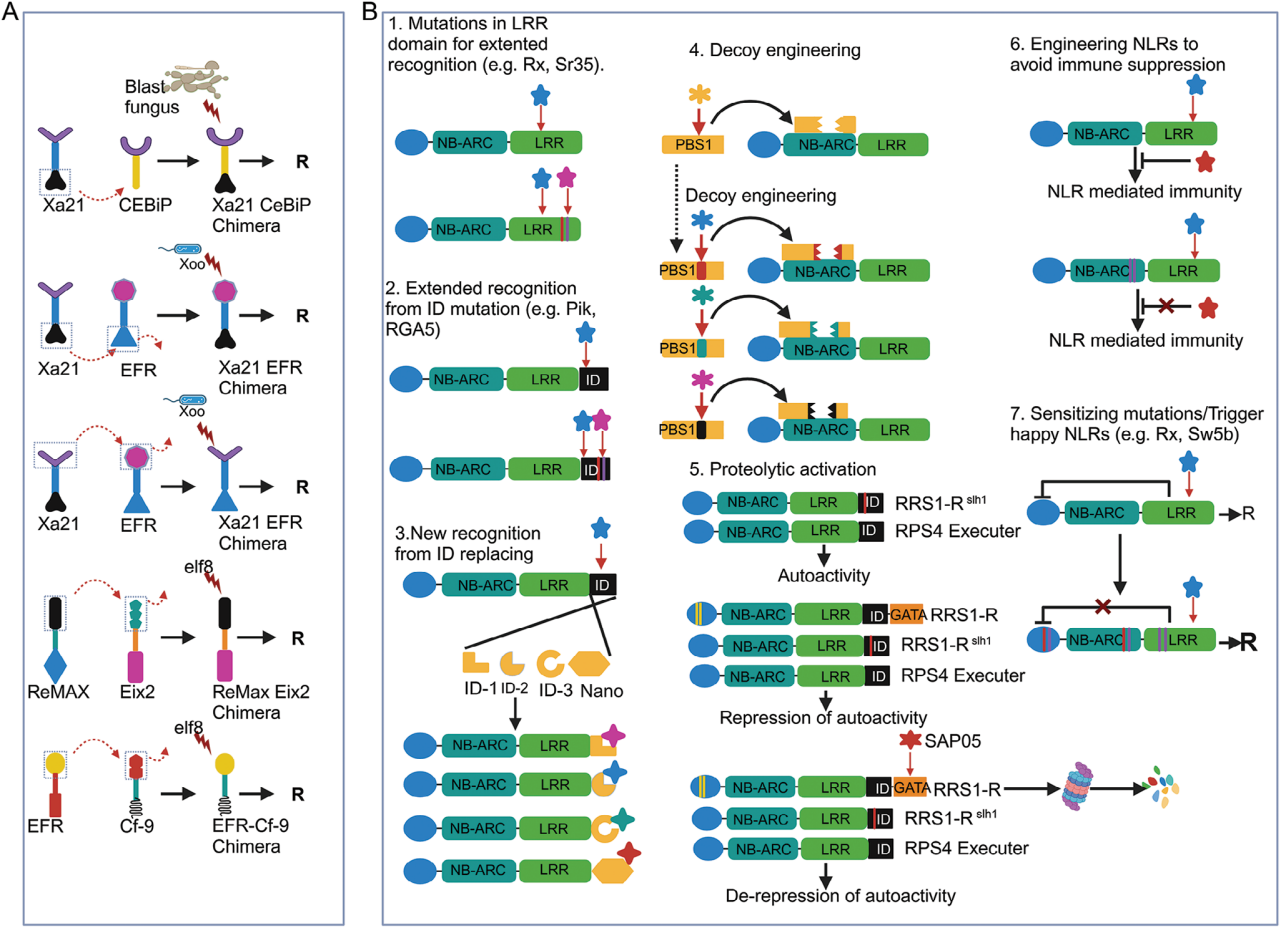
Strategies to engineer immune receptors for developing resistance in plants. A) Different examples of PRR engineering in which chimeric proteins obtained from swapping and recombining different domains, provided functional resistance in plants. B) Multiple approaches adopted for NLR engineering focus on modifying or extending effector recognition specificity or enhancing NLR activation sensitivity. The approaches used for modifying or extending effector recognition specificity include, mutations in LRR domains enhancing NLR recognition 1), mutations in the integrated domains (ID) for extended recognition 2) or creating new recognition form ID replacement with other IDs or nanobodies 3), targeting guardee/decoy system by replacing their cleavage sites for other protease effectors for engineering in novel disease resistance specificities 4), creating a synthetic NLR circuit, where effector presence or absence, by proteolytic activation, controls the induction of NLR‐based immunity 5) and engineering NLRs to avoid immune suppression, for example, helper NLR, NRC2 that was initially immunosuppressed by SS15 from potato cyst nematode, was engineered to escape SS15 mediated suppression 6). In addition to enhancing the recognition capacity of NLRs for effector, enhancing NLRs activation sensitivity by introducing “trigger happy” mutations in NLRs can result in a lower activation threshold thus improving recognition by NLRs 7).

Plant NLRs are the intracellular immune receptors that exhibit conserved domain architecture, carrying a central nucleotide‐binding (NB‐ARC) domain, a C‐terminal leucine‐rich repeat (LRR) domain and an N‐terminal signaling domain. The N‐terminal domain is either a Toll/Interleukin‐1/Receptor (TIR) domain (in TNL NLRs) or coiled‐coil (CC) domain (in CNL NLRs).^[^ ^64^ ^]^ Because of their major roles in disease resistance, NLRs are popular for breeding disease resistance in crops.^[^ ^65^ ^]^ In order to avoid host immunity, the pathogens use different strategies to truncate or delete effectors, block the expression of effectors, or evolve new effector variants through mutagenesis that cannot be recognized by the NLRs.^[^ ^66^ ^]^ New NLR engineering methods aimed to avoid these limitations or to broaden effector recognition specificities are emerging. Multiple approaches adopted for NLRs engineering described in the following sections focus on modifying or extending effector recognition specificity, enhancing NLRs activation sensitivity and engineering NLRs to avoid immune suppression (**Figure** **3**) (Table 2).

##### Modified or Extended Effector Recognition Specificity

In many NLRs, the LRR domain is a well‐characterized domain for effector recognition. Random or targeted mutations in the LRR domain can extend disease‐resistance specificity or result in novel recognition specificities. For example, random mutations in R protein (Rx) extended disease‐resistance specificity against previously unknown *Potato virus X* and *Poplar mosaic virus* (PopMV) strains.^[^ ^67^ ^]^ In another example, targeted mutagenesis within the LRR domains of two related Sr35 homologs (Sh), TaSh1 in bread wheat and HvSh1 in barley, using the structural information of the wheat NLR Sr35, created their bioengineered versions that were able to detect AvrSr35, which previously was not recognized in these plants.^[^ ^68^ ^]^ Similarly, in another study, the structural information of wheat Sr50 was used to engineer the LRR domain of wheat Sr33 to gain the recognition of *Pgt* effector AvrSr50.^[^ ^69^ ^]^

Many NLRs also contain non‐canonical integrated domains (IDs) that resemble effector targets. The integrated domains can directly bind to the effectors or are modified by effectors, thereby initiating ETI.^[^ ^70^ ^]^ Enhanced or novel recognition specificities can also be obtained by the introduction of targeted mutations into the integrated domains.^[^ ^71^ ^]^ For example, engineering the rice NLR pair, Pik (Pik‐1/Pik‐2) produced a novel Pik allele that bound to previously unrecognized variants of AVR‐Pik.^[^ ^72^ ^]^ Similarly, the NLR pair, RGA5/RGA4, was engineered by introducing targeted mutations in the HMA domain of RGA5 to enable interaction with Avr‐PikD in tobacco.^[^ ^73^ ^]^ However, the transgenic rice plants did not exhibit disease resistance against *M. oryzae*. In another example, engineering the binding interface of the rice RGA5‐HMA domain enabled it to recognize a non‐corresponding MAX effector, Avr‐Pib, providing resistance to the *M. oryzae* isolates expressing *AvrPib*.^[^ ^88^ ^]^ Yet in another example, a variant of RGA5‐HMA, by mutating residues within and outside the HMA domain, was created in rice which bound to noncorresponding effector AVR‐PikD, conferring resistance to the *M. oryzae*.^[^ ^74^ ^]^

Novel recognition specificities can also be created by replacing integrated domains of NLRs with other effectors targets, guardees/decoys, or nanobodies. For example, transgenic rice with novel resistance toward stealthy AVR‐Pik variants was created by replacing the Pik‐1 HMA domain with the structure of OsHIPP19, a natural host target.^[^ ^75^ ^]^ In another study, swapping the Pik‐1 HMA domain with fragments of single‐chain antibodies called nanobodies that bind fluorescent proteins (FPs), triggered ETI and conferred resistance against the plant viruses that expressed the corresponding FPs.^[^ ^76^ ^]^

Many NLRs perceive effectors indirectly through modifications of effector targets called guardee or the mimics of true host targets called decoy. The guardee/ decoy system can also be engineered to develop resistance to a specific pathogen effector. For example, targeted modification of cleavage site of *Pseudomonas syringae* protease effector AvrPphB in AvrPphB SUSCEPTIBLE 1 (PBS1) to the cleavage sequences that were recognized by the Nla protease from *turnip mosaic virus* (TuMV) or from *tobacco etch* virus (TEV) switched the RPS5 recognition specificity to them.^[^ ^77^ ^]^ PBS1‐based decoy engineering has been successfully used to engineer resistance to soybean mosaic virus (SMV)^[^ ^78^ ^]^ or potato virus Y (PVY)^[^ ^79^ ^]^ in soybean and potato respectively.

Resistance can also be engineered by creating a synthetic NLR circuit, where effector presence or absence controls the induction of NLR‐based immunity. This can be applied to NLRs that occur in pairs in such a way that the sensor NLR responds to pathogen effectors for activation. For instance, the TIR‐NLRs pair, RRS1‐R/RPS4, was engineered to respond to a phytoplasma effector, SAP05, by adding a SAP05‐dependent degron domain to the C‐terminus of RRS1‐R. This activated a defense response in the presence of SAP05 in tobacco transient expression assays.^[^ ^80^ ^]^ Although the transgenic Arabidopsis did not exhibit complete resistance to phytoplasma infection, this approach can provide a new strategy to develop novel effector recognition specificities against many phytopathogens and engineer disease resistance in plants. In addition, this can also be a good way to overcome autoimmunity caused by the engineered NLR.

##### Enhanced NLR Activation Sensitivity (Trigger‐Happy NLRs)

Recognition of effector by NLR may not be always sufficient, as an effective defense response also relies on the subsequent activation of immune signaling. For example, the rice Pikp‐1 HMA domain is able to bind to three different AVR‐Pik variants. However, disease resistance is produced only when the HMA domain of Pikp‐1 interacts with AVR‐PikD.^[^ ^70^, ^81^ ^]^ Compared to the strength of HMA domain interaction with other variants, interaction with AVR‐PikD variant is at least ten times stronger, suggesting that for an efficient disease response, the stability of NLR‐effector complex must exceed a specific threshold. These findings pave the way to enhance NLR activation sensitivity and make “trigger‐happy” NLRs with reduced thresholds of activation. “Trigger‐happy” NLRs can be obtained by inducing mutations across conventional NLR domains including the relatively conserved NB and CC domains.^[^ ^71^, ^82^ ^]^ An example of trigger‐happy NLRs is sensitizing an *Rx* mutant that originally displayed a delayed response to *poplar mosaic virus* (PoMV).^[^ ^67^ ^]^ Using random mutagenesis, a sensitized form of *Rx* with one to two amino acid substitutions in the NB‐ARC domain, provided full immunity towards PoMV.^[^ ^83^ ^]^ In another example, using a stepwise artificial evolution involving a sensitizing mutation in the LRR domain, an engineered version of tomato Sw5b NLR was created. The sensitized Sw5b provided resistance against resistance‐breaking isolates of *tomato spotted wilt virus* (TSWV).^[^ ^84^ ^]^ Potentially, improved NLRs can be obtained by enhancing their effector recognition specificities and incorporating trigger‐happy mutations with less activation threshold in them.^[^ ^82^ ^]^

##### Engineering NLRs to Avoid Immune Suppression

Because of their important role in plant immunity, NLRs are often targeted by pathogen effectors in an ongoing evolutionary battle.^[^ ^85^ ^]^ Resistance can also be developed by engineering such NLRs to avoid immune suppression by pathogen effectors. For example, the potato cyst nematode (*Globodera rostochiensis*) secretes SPRYSEC15 (SS15) effector which binds to the central NB‐ARC domain, HD1‐1, of tomato helper NLR NRC1 and tobacco helper NLRs (NRC2 and NRC3), inhibiting downstream signaling. In contrast, SS15 cannot suppress NRC4, an NRC2/3 paralog from tobacco.^[^ ^85^ ^]^ Based on NRC4's resilience to SS15 inhibition, engineering the binding site of SS15 in NRC2 caused NRC2 to evade suppression by SS15 in tobacco, restoring the expression of numerous disease‐resistance genes.^[^ ^86^ ^]^ This work describes that NLR engineering to avoid immune suppression can also be potentially used to achieve robust immunity and avoid parasite suppression.

#### 3.2.3 Limitations of Engineering Resistance Using Immune Receptors and Proposed Solutions

##### PRRs

A major concern of introducing foreign PRRs or engineering PRRs is that this might negatively impact the association of the recipient plants with beneficial and/or symbiotic microbes. A lack of genetic and molecular adaptations between the interacting partners often results in the termination of mutualistic interactions.^[^ ^90^ ^]^ Therefore, the effect of foreign PRRs on the microbiome should be evaluated in transgenic plants before taking them to the field.^[^ ^91^ ^]^ *Medicago truncatula* plants transgenically expressing receptor EFR from Arabidopsis showed increased resistance to the bacterial root pathogen, *Ralstonia solanacearum* which infects a large number of plant species. In addition, compared to the wild‐type plants, the transgenic plants showed delayed nodule formation but the final extent of nodulation and nitrogen fixation was not affected.^[^ ^91^ ^]^ This suggests that adopted symbionts have evolved multiple ways to evade or suppress host PTI at different levels.^[^ ^92^ ^]^ The problems associated with heterologous PRR expression can be potentially solved by PRR modification at the pathogen or PAMP epitopes binding site, instead of introducing a foreign PRR or a foreign domain (in the case of chimeric PRRs).^[^ ^93^ ^]^ In order to get recognition for a particular epitope variant, the ligand binding sites can be subjected to in vitro evolution^[^ ^94^ ^]^ or potentially to directed modification using tools such as CRISPR/Cas‐mediated genome editing. Another potential method is to design PRRs with the help of artificial intelligence (AI) tools using the structural information of PAMPs/DAMPs from pathogens and PRRs from plants. Another problem with PRR transfer/engineering is using constitutive promoters for the over‐expression of heterologous PRRs that can create growth abnormalities. This problem can be fixed by using plant promoters from endogenous PRRs to drive heterologous PRRs expression in plants.^[^ ^95^ ^]^

##### NLRs

A bottleneck to producing plants resistant to disease through NLR engineering is the resulting autoimmune phenotypes, where the plant immune response is continuously active in the absence of effectors which is deleterious to the health of plants. The auto‐immunity may result from the incompatible combinations in the highly complex NLR signaling networks that coevolve together.^[^ ^4^ ^]^ Choosing the right downstream partner NLR mitigates the auto‐activity as demonstrated for the rice NLR pair Pik (sensor NLR Pik1/ helper NLR Pik2).^[^ ^96^ ^]^ Additionally, using the detailed structural information of the interaction between effectors and NLRs, and introducing amino acids polymorphism in the newly integrated domain can reduce autoimmunity.^[^ ^68^, ^72^, ^75^ ^]^ Moreover, choosing from a population of NLRs and/or NLR regions subjected to artificial evolution or using simple NLRs with less downstream partners would also solve the problem of autoimmunity and improve the efficiency of engineering NLRs.^[^ ^97^ ^]^

Autoimmunity can also be overcome by employing transgene‐cassette containing *NLR* genes driven by pathogen‐specific, inducible promoters as applied to other defense‐related genes.^[^ ^98^ ^]^ Another strategy, effective against those pathogens that release TALEs, is precisely editing the promoters of the newly introduced NLRs to provide binding sites for the TALEs, making pathogen inducible expression of the introduced NLRs. In a recent study, it was found that NLRs maintain a balanced immune response without auto‐activity through oligomerization at high concentrations, and binding of inositol phosphates (IP) as cofactors. Inducing mutations at the IP binding sites abolished the balanced immune response.^[^ ^99^ ^]^ Targeted regulation of post‐translation concentrations of foreign NLRs will create more opportunities to avoid auto‐activity associated with them.

### 3.3 Engineering Components of Systemic Acquired Resistance (SAR) in Plants

Components of SAR, which rely on SA and Pip/NHP, can also be manipulated for engineering disease resistance in plants.^[^ ^1^ ^]^ SA‐dependent response is mediated by NPR protein family members, NPR1, NPR3, and NPR4 that serve as co‐receptors of SA.^[^ ^100^ ^]^ Briefly, the binding of SA to NPR1 causes its activation which then positively regulates the expression of *PR* genes.^[^ ^101^ ^]^ In contrast, NPR3/4 inhibits pro‐immune genes, such as *SARD1, WRKY70*, and *TFs* in the TGA protein family, acting as negative regulators when SA is not available. Pathogen attack induces SA that binds and suppresses the repressor activity of NPR3/4, which then together with NPR1, positively regulates pro‐immune genes.^[^ ^100^, ^102^ ^]^

Resistance to a variety of pathogens has been developed by over‐expressing Arabidopsis* NPR1* in many plant families.^[^ ^103^ ^]^ However, continuous induction of defense response due to constitutive *NPR1* expression is associated with fitness problems. Employing a stringent mechanism involving the expression of *NPR1* under “*TBF1‐cassette*”, consisting of an immune‐inducible promoter as well as two pathogen‐responsive upstream open reading frames from the TBF1 gene (*uORFs_TBF1_*), produced resistance to rice blast without a notable yield penalty.^[^ ^98^, ^104^ ^]^ Suppressing *NPR3/4* that act as negative regulators in SAR pathway with stringent transcription and translational regulation can also be used for disease resistance in plants.

NHP has lately been identified as an essential immune signal for SAR.^[^ ^105^ ^]^ The mutants of three highly conserved enzymes, ALD1, SARD4, and FMO1, involved in NHP biosynthesis, are compromised in SAR.^[^ ^1^ ^]^ Reinforcing the NHP pathway may therefore develop disease resistance in plants. Transiently overexpressing Arabidopsis NHP biosynthesis enzymes, *ALD1*, *SARD4*, and *FMO1*, in tomato or NHP treatment of sweet pepper induced SAR and protected the plants from infection at remote sites.^[^ ^106^ ^]^ Moreover, in many monocots and dicots, the biosynthesis of NHP, upon exposure to pathogen, induces SAR.^[^ ^107^ ^]^ This indicates that NHP has a consistent function in the establishment of SAR in plants. Modification of NHP biosynthesis to improve SAR in a regulated manner thus can act as a useful strategy to develop disease resistance in plants.

PR proteins and antimicrobial peptides (AMPs) are a group of diverse molecules induced by phytopathogens and by defense‐related signaling molecules. They play a key role in plant immune system, especially during SAR. Resistance engineering against a variety of pathogens by the overexpression of *PR* genes or *AMP* genes has been one of the popular choices for researchers.^[^ ^108^ ^]^ For example, increased resistance to powdery mildew infection was obtained by the overexpression of *PR4* gene in *Vitis vinifera*.^[^ ^109^ ^]^ Transgenic tobacco and maize exhibited enhanced pathogen resistance and salt tolerance by overexpressing the *LcCHI2 chitinase* gene from *Leymus chinensis* (false wheatgrass) in them.^[^ ^110^ ^]^ As a further example, in soybean, increased resistance to Phytophthora root and stem rot caused by *Phytophthora sojae* (*P. sojae*) was obtained, by the over‐expression of *CaAMP1* gene.^[^ ^111^ ^]^ Moreover, enhanced resistance to leaf rust pathogen *Puccinia triticina* was obtained by the overexpression of a *M. truncatula AMP defensin* gene (*MtDef4.2*) in wheat without affecting the colonization of beneficial arbuscular mycorrhizal fungus *Rhizophagus irregularis*, in transgenic wheat.^[^ ^112^ ^]^ Besides the success, there have been some reports where no resistance against pathogens was obtained by *PR* transgenic technology.^[^ ^108^ ^]^ Since the overexpression of *PR* genes or AMPs often confers quantitative resistance, and given the evolutionary changes in the corresponding pathogen *Avr* gene over time, the function of a single *PR* gene may change. This problem can be addressed by stacking multiple *PR* genes into the host plant.^[^ ^5^ ^]^

The key regulatory factors in SAR pathway, including the mechanism that how PTI and ETI signals activate SA synthesis, is not clear. Our increasing understanding of the SA signaling pathway and its interactions with other immune signaling sectors may lead to improved strategies for engineering SAR for plant disease management. The mobile signal for initiating SAR in distal tissue has recently been identified.^[^ ^113^ ^]^ Manipulating this mobile signal, and exploring other mobile signals for SAR will open up new possibilities for engineering disease resistance in plants. Plants and animals have several disease‐associated SA targets in common,^[^ ^114^ ^]^ suggesting that the findings from animals research on SA can be integrated into plant disease management.

### 3.4 Overexpression of Positive Regulators of Plant Immunity

Recognition of pathogens by immune receptors triggers an array of dynamic and interconnected signaling events that are tightly regulated by various mechanisms and components, including calcium signaling, transcriptional regulators, mitogen‐activated protein kinase (MAPK/MPK) cascades, plant hormones, and microRNAs. Resistance can be engineered by enhancing the activity of the positive regulators if the pleiotropic effects can be avoided. For example, the transcription factor *WRKY45* plays a key role in the SA signaling pathway and strengthens PTI response against *Xoo* and *M. oryzae* in rice.^[^ ^115^ ^]^ Rice with enhanced resistance to both *Xoo* and *M. oryzae* was obtained by the over‐expression of *WRKY45* under the maize *ubiquitin* promoter but the transgenic plants showed growth defects.^[^ ^115^ ^]^ However, the optimized expression of *WRKY45* by testing 16 constitutive promoters of different strengths produced rice with enhanced resistance and normal agronomic traits in the field.^[^ ^116^ ^]^ The transient elevation of cytoplasmic calcium is essential for plant immunity. In rice, a cyclic nucleotide‐gated channel protein, OsCNGC9, positively regulates the resistance to blast disease by mediating PAMP‐induced calcium influx. Consequently, the overexpression of *OsCNGC9* conferred enhanced blast resistance in rice.^[^ ^117^ ^]^ MAPK/MPK cascades are key signaling modules that play roles in signal transduction during PTI and ETI as well as during SAR.^[^ ^118^ ^]^ The overexpression of *MPKK10.2* in rice promoted *Xoc* resistance and drought tolerance by activating different MAPKs.^[^ ^119^ ^]^

Plant hormones including SA, jasmonic acid (JA), and ethylene (ET), play crucial roles in plant response to pathogens. SA (described in SAR section) is involved in activating defense signaling against a variety of biotrophic and hemibiotrophic pathogens, while JA and ET are thought to be associated with defense responses against necrotrophic and hemibiotrophic pathogens.^[^ ^120^ ^]^ OsbHLH034, a bHLH‐type transcription factor that plays a key role in the JA‐mediated defense response, enhanced resistance against *Xoo* when overexpressed in rice.^[^ ^121^ ^]^ The *ETHYLENE RESPONSE FACTOR* (*ERF*) gene *ERF96* provided resistance to the bacterial pathogen *Pectobacterium carotovorum* and necrotrophic fungal pathogen *Botrytis cinereal*, when overexpressed in Arabidopsis.^[^ ^122^ ^]^ One of the rate‐limiting enzymes in ethylene biosynthesis, *OsACS2*, when overexpressed in rice, provided resistance to *Rhizoctonia solani* and *M. oryzae*.^[^ ^123^ ^]^ The overexpression of ethylene signaling components, *OsEIN2* and *OsEIL1* activated JA biosynthesis and the subsequent phytoalexin accumulation in transgenic rice, providing resistance to *M. oryzae* infection.^[^ ^124^ ^]^ Plants have also developed RNA components, especially the recently identified *microRNAs* (*miRNAs*) counter disease challenges.^[^ ^125^ ^]^ Broad‐spectrum resistance to fungal and bacterial pathogens was obtained by the overexpression of *Osa‐miR160*, partially by suppressing *Auxin Response Factor 8 (ARF8)* in rice.^[^ ^126^ ^]^

Like immune receptors, one problem associated with overexpressing some positive regulators of immunity is autoimmunity which affects plant growth. Employing pathogen‐inducible promoters can help to solve the autoimmunity problem.

### 3.5 Genome Editing of *S* Genes for Resistance Breeding

**Figure 4:**
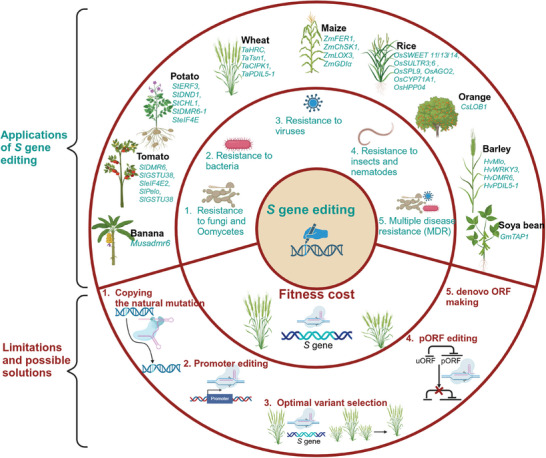
*S* gene editing: applications, limitations and proposed solutions. *S* gene editing is used to develop resistance against fungi/oomycetes, bacteria, viruses, insects, and nematodes in important crops such as wheat, maize, rice, barley, tomato, potato, soybean, banana, and orange. The complete loss of function of *S* genes often leads to fitness cost. The fitness cost associated with *S* gene editing can be solved by various methods such as copying the natural mutation, promoter editing, optimal variant section from a population of CRISPR mutants, Upstream ORFs (uORFs) editing, or de novo uORFs making.

Although developing resistance in plants using dominant immune receptor genes or positive regulators is popular because of their complete resistance to a set of pathogens and easy selection in breeding programs, the resistance conferred by immune receptors is not long‐lasting due to repaid mutations and changes in pathogens virulence. An alternative strategy that offers a solution to overcome the limitations of immune receptors for resistance breeding is the disruption of the function of *S* genes, the host genes that are manipulated by pathogens for successful infection.^[^ ^6^ ^]^ *S* gene editing has been used against a variety of phytopathogens including fungi and oomycetes, bacteria, viruses, insects pests and nematodes (**Figure** **4**). For detailed list of *S* genes, please refer to **Table** **3**.

**Table 3:** *S* genes used for disease resistance in plants.

Editing *S* genes for resistance to filamentous pathogens (fungi and oomycetes)						
Gene name	Method	Trans‐gene free (Yes/No)	Plant Species	Pathogen	Disease	Reference
*MdMLO19*	RNAi	No	*Malus Domestica*	*Podosphaera leucotricha*	Powdery mildew	[150]
*GbMPK3*	RNAi	No	Cotton	*Verticillium dahliae*	Verticillium wilt	[150]
*GmTAP1*	CRISPR/Cas9	Yes	*Glycine max*	*Phytophthora sojae*	Root rot	[151]
*HvCRK1*	RNAi	No	*Hordeum vulgare*	*Blumeria graminis* f. sp. *hordei*	Powdery mildew	[152]
* OsDCL1*	RNAi	No	*Oryza sativa*	*Magnaporthe oryzae*	Rice blast	[153]
*OsDjA6*	RNAi	No	*Oryza sativa*	*Magnaporthe oryzae*	Rice blast	[154]
*OsWRKY28*	T DNA	No	*Oryza sativa*	*Magnaporthe oryzae*	Rice blast	[155]
*OsERF922*	RNAi	No	*Oryza sativa*	*Magnaporthe oryzae*	Rice blast	[156]
*OsCPK18*	RNAi	No	*Oryza sativa*	*Magnaporthe oryzae*	Rice blast	[157]
*Osa‐miR1873*	Target mimicry	No	*Oryza sativa*	*Magnaporthe oryzae*	Rice blast	[158]
*OsVOZ1* and *OsVOZ2*	T‐DNA, CRISPR/ Cas9, RNAi	No	*Oryza sativa*	*Magnaporthe oryzae*	Rice blast	[159]
*OsCPK18/* *OsCPK4*	CRISPR/ Cas9	Yes	*Oryza sativa*	*Magnaporthe oryzae*	Rice blast	[160]
*BnCRT1a*	CRISPR/ Cas9, EMS	Yes	*Brassica napus*	Verticillium longisporum	Verticillium stripe	[161]
*SlMlo1*	CRISPR/ Cas9	No	*Solanum lycopersicum*	Oidium neolycopersici	Powdery mildew	[142]
*StERF3*	CRISPR/ Cas9	Yes	*Solanum tuberosum*	*Phytophthora infestans*	Potato late blight	[162]
*StDND1, StCHL1, *and *DMG400000582 (StDMR6‐1)*	CRISPR/ Cas9	Yes	*Solanum tuberosum*	*Phytophthora infestans*	Potato late blight	[163]
*StDND1, StCHL1, StDMR6‐1*	CRISPR/ Cas9	Not confirmed	*Solanum tuberosum*	*Phytophthora infestans*	Potato late blight	[163]
*StERF3*	RNAi	No	*Solanum tuberosum*	*Phytophthora infestans*	Potato late blight	[164]
*StERF3*	RNAi	No	*Solanum tuberosum*	*Phytophthora infestans*	Potato late blight	[164]
* FaWRKY25*	RNAi	No	*Fragaria × ananassa* (Benihoppe)	Botrytis cinerea	Gray mold	[165]
*TaHRC*	CRISPR/ Cas9	Yes	*Triticum aestivum*	*Fusarium graminearum*	Fusarium head blight (FHB)	[164]
*TaHRC*	Natural recessive allele	Yes	*Triticum aestivum*	*Fusarium graminearum*	FHB	[131]
*TaCIPK14*	CRISPR/ Cas9	Yes	*Triticum aestivum*	Puccinia striiformis f. sp. tritici	Stripe rust	[166]
*TaDIR‐B1*	EMS	Yes	*Triticum aestivum*	Fusarium pseudograminearum	Fusarium crown rot	[167]
*TaCSN5*	RNAi	No	*Triticum aestivum*	Puccinia striiformis f. sp. tritici	Stripe rust	[168]
*TaEDR1*	CRISPR/ Cas9	Yes	*Triticum aestivum*	Puccinia graminis f. sp. tritici	Powdery mildew	[169]
*TaPsIPK1 *	CRISPR/ Cas9	Yes	*Triticum aestivum*	Puccinia striiformis f. sp. tritici	Stripe rust	[170]
*ZmFER1*	CRISPR/ Cas9	Yes	*Zea mays*	*Fusarium verticillioides*	Ear rot	[134]
*ZmFBL41*	Transposon‐insertion	No	*Zea mays*	*Rhizoctonia solani*	Leaf and sheath blight	[171]
*ChSK1 *	CRISPR/ Cas9	Not confirmed	*Zea mays*	*Cochliobolus heterostrophus*	Southern leaf blight (SLB)	[172]
*ZmLOX3*	CRISPR/ Cas9	Yes	*Zea mays*	*Ustilago maydis*	Corn smut	[173]
*Editing of S genes for resistance to bacteria*						
*CsDMR6 *	CRISPR/ CAS9	Transgenic	“Duncan” grapefruit and Carrizo citrange	*Xanthomonas citri* subsp. *citri*	Citrus canker	[174]
*MusaDMR6*	CRISPR/ Cas9	No	Banana	*Xanthomonas campestris* pv. *musacearum*	Banana Xanthomonas wilt (BXW)	[175]
*LOB1*	CRISPR‐SpCas9p	No	Grapefruit	*Xanthomonas citri* subsp. *citri*	Citrus canker	[176]
*CsLOB1*	CRISPR‐SpCas9p	No	Grapefruit	*Xanthomonas citri* subsp. *citri*	Citrus canker	[177]
*LOB1*	CRISPR/ SpCas9p	No	Pummelo (*Citrus maxima*)	*Xanthomonas citri* subsp. *citri*	Citrus canker	[178]
*OsSWEET11, OsSWEET13*, and *OsSWEET14*	CRISPR/ Cas9	Yes	*Oryza sativa*	Xanthomonas oryzae pv. oryzae	Bacterial blight (BB)	[137]
*Os8N3*	CRISPR Cas9	Yes	*Oryza sativa*	Xanthomonas oryzae pv. oryzae	BB	[179]
*OsSULTR3;6*	CRISPR/ Cas9	Yes	*Oryza sativa*	Xanthomonas oryzae pv. oryzae	Bacterial leaf streak (BLS)	[180]
*OsSWEET14*	TALEN	Yes	*Oryza sativa*	Xanthomonas oryzae pv. oryzae	BB	[181]
*DEP1*	RNAi, T‐DNA	No	*Oryza sativa*	Rhizoctonia solani		[182]
*OsSWEET13*	CRISPR/ Cas9	No	*Oryza sativa*	Xanthomonas oryzae pv. oryzae	BB	[183]
*GF14e*	RNAi	No	*Oryza sativa*	Xanthomonas oryzae pv. oryzae	BB	[184]
*OsNramp6*	T‐DNA	No	*Oryza sativa*	*Magnaporthe oryzae*	Rice blast	[185]
*OsHCAR*	CRISPR/ Cas9	Not confirmed	*Oryza sativa*	Xanthomonas oryzae pv. oryzae	BB	[186]
* OsPG1*	CRISPR‐Cas9	No	*Oryza sativa*	Xanthomonas oryzae pv. oryzae	BB	[187]
*OsSWEET11* and *OsSWEET14*	CRISPR/ Cas9	No	*Oryza sativa*	Xanthomonas oryzae pv. oryzae	BB	[137]
*OsSULTR3;6, OsSWEET11* and *OsSWEET14 *	CRISPR/ Cas9	Yes	*Oryza sativa*	Xanthomonas oryzae pv. oryzae, Xanthomonas oryzae pv. oryzicola	BB, BLS	[188]
*WRKY22*	RNAi	No	*Citrus sinensis*	*Xanthomonas citri* subsp*. citri*	Citrus canker	[189]
*SlSRFR1*	CRISPR/ Cas9	No	*Solanum lycopersicum*	*Pto DC3000*	Bacterial speck disease	[190]
*CsLOB1*	CRISPR‐Cas9	No	Wanjincheng orange	*Xanthomonas citri* subsp. *citri*	Citrus canker	[191]
Musadmr6	CRISPR‐Cas9	No	Banana	*Xanthomonas campestris* pv. *musacearum*	BXW	[175]
*OsMAPK6*	CRISPR‐Cas9	Not confirmed	*Oryza sativa*	*Xanthomonas oryzae* pv. *oryzicola*	BLS	[53]
*OsEDR1*	RNAi, T DNA	No	*Oryza sativa*	Xanthomonas oryzae pv. oryzae	BB	[192]
*Editing of S genes for resistance to viruses*						
*Hv*PDIL5‐1	Naturally occurring recessive resistance locus rym11	Yes	*Hordeum vulgare*	*Barley yellow mosaic virus* (BaYMV) and *barley mild mosaic virus* (BaMMV)	Barley yellow mosaic virus disease	[193]
eIF4E	CRISPR‐Cas9	Yes	Cucumber	Zucchini yellow mosaic virus (ZYMV) and Papaya ring spot mosaic virus‐W (PRSV‐W)	Mosaic diseases	[141]
*ZmGDIα*	Natural variant	Yes	*Zea mays*	*Rice black‐streaked dwarf virus* (RBSDV)	Maize rough dwarf disease (MRDD)	[194]
*ZmGDIα*	CRISPR/ Cas9	Yes	*Zea mays*	*Rice black‐streaked dwarf virus* (RBSDV)	Maize rough dwarf disease (MRDD)	[134]
*OsSPL9*	CRISPR/ Cas9	No confirmed	*Oryza sativa*	*Rice stripe virus* (RSV)	Rice stripe disease	[195]
*OsAGO2*	CRISPR/ Cas9	No	*Oryza sativa*	*Rice black‐streaked dwarf virus* (RBSDV)	Maize rough dwarf disease (MRDD)	[196]
*SlGSTU38*	CRISPR/ Cas9	Yes	*Solanum lycopersicum*	*Pepino mosaic virus* (PepMV)	Pepino mosaic disease	[197]
eIF4E2	EMS	Yes	*Solanum lycopersicum*	*Pepper veinal mottle virus* (PVMV)	Leaf mosaic disease	[142]
*Pelo*	CRISPR/ Cas9	No	*Solanum lycopersicum*	*Tomato yellow leaf curl virus* (TYLCV),	Yellow leaf curl disease	[142]
PDIL5‐1	CRISPR/ Cas9	Not confirmed	*Triticum aestivum*	*Wheat yellow mosaic virus* (WYMV)	Wheat yellow mosaic disease	[198]
*Editing of S genes for resistance to insects and nematodes*						
*CYP71A1*	CRISPR/ Cas9	Not confirmed	*Oryza sativa*	Rice brown planthopper (BPH)		[143]
OsHPP04	CRISPR/ Cas9	Yes	*Oryza sativa*	Meloidogyne graminicola		[145]
*Editing of S genes for multiple disease resistance (MDR)*						
Multiplex editing of *Pi21, Bsr‐d1* and *Xa5*	CRISPR/ Cas9,	No	*Oryza sativa*	*Magnaporthe oryzae, Xanthomonas oryzae pv. oryzae*	Rice blast, BB	[149]
*SlDMR6‐1*	CRISPR/ Cas9	No	*Solanum lycopersicum*	*Pseudomonas syringae* pv. *tomato*, *Xanthomonas gardneri*, *Xanthomonas perforans*, Phytophthora capsica, Pseudoidium neolycopersici	Bacterial speck, bacterial spot, footrot, powdery mildew	[148]
*StDND1*	RNAi	No	*Solanum tuberosum*	Phytophthora infestans, Oidium neolycopersici, Golovinomyces orontii	Potato late blight, powdery mildew	[199]
SlDND1	RNAi	No	*Solanum lycopersicum*	Phytophthora infestans, Oidium neolycopersici, Golovinomyces orontii	Potato late blight, powdery mildew	[199]
*OsGLIP1 *and * OsGLIP2*	RNAi	No	*Oryza sativa*	*Magnaporthe oryzae, Xanthomonas oryzae pv. oryzae*	BB, Rice Blast	[200]
*SPL33*	EMS	No	*Oryza sativa*	*Magnaporthe oryzae, Xanthomonas oryzae pv. oryzae*	BB, Rice Blast	[201]
*OsMADS26‐*	RNAi	No	*Oryza sativa*	*Magnaporthe oryzae, Xanthomonas oryzae pv. oryzae*	BB, Rice Blast	[202]
*OsMPK15*	CRISPR/ Cas9	Not confirmed	*Oryza sativa*	*Magnaporthe oryzae, Xanthomonas oryzae pv. oryzae*	BB, Rice Blast	[203]
*TMS5, Pi21*, and *Xa13*	CRISPR/ Cas9	Yes	*Oryza sativa*	*Magnaporthe oryzae, Xanthomonas oryzae pv. oryzae*	BB, Rice Blast	[204]
*OsTrxm*	CRISPR/ Cas9	No	*Oryza sativa*	Xanthomonas oryzae pv. oryzae, Rhizoctonia solani	BB and Sheath blight	[205]
*LMR*	RNAi, EMS	No	*Oryza sativa*	*Magnaporthe oryzae, Xanthomonas oryzae pv. oryzae*	BB, Rice blast	[206]
*HDT701*	RNAi	No	*Oryza sativa*	*Magnaporthe oryzae, Xanthomonas oryzae pv. oryzae*	BB, Rice blast	[207]
*OsMESL*	T‐DNA, RNAi, CRISPR/ Cas9	No	*Oryza sativa*	*Magnaporthe oryzae, Xanthomonas oryzae pv. oryzae*, Rhizoctonia solani	BB, Sheath blight, Rice blast	[205]
*OsCUL3a*	EMS	Non	*Oryza sativa*	M oryzae, Xanthomonas oryzae pv. oryzae	Rice blast, BB	[208]
*OsCPK4 *	T DNA	No	*Oryza sativa*	M oryzae, Xanthomonas oryzae pv. oryzae	Rice blast, BB	[209]
*Microrchidia MORC1 and MORC6a*	CRISPR/ SpCas9	Not confirmed	*Hordeum vulgare*	*B. graminis, Fusarium graminearum*	Powdery mildew and FHB	[210]

An important example is a recessive mutant of barley gene, *Mutation‐induced recessive alleles* (*Mlo*) which was reported nearly eight decades ago to show resistance to powdery mildew and still provides a major resistance source in the field against all powdery mildew races.^[^ ^127^ ^]^ *Mlo*, which encodes a membrane‐associated protein having seven transmembrane domains, is conserved in monocots and dicots. It is required for the penetration of powdery mildew fungus to the host epidermal cells, thus establishing a successful infection.^[^ ^128^ ^]^ CRISPR/Cas9 mediated mutation of *Mlo* orthologues in different species, including wheat^[^ ^129^ ^]^ and tomato,^[^ ^129^ ^]^ has been used to confer resistance to powdery mildew in them. Another example is resistance to Fusarium head blight (FHB) that is a fungal disease of cereals crops particularly wheat and barley, causing significant yield losses. Several quantitative trait loci (QTLs) and genes are associated with FHB resistance. However, *Fhb1* identified in Sumai 3, a Chinese wheat cultivar, has an exceptional and consistent effect on FHB resistance.^[^ ^130^ ^]^ The *Fhb1*‐based resistance in wheat was found to be due to the loss of function mutation spanning the start codon of the *TaHRC*.^[^ ^131^ ^]^ Consistently, using biolistic transformation, *TaHRC* mutation through CRISPR/Cas9, increased FHB resistance in the susceptible wheat cultivar, indicating that this approach can be effectively applied in certain genetic backgrounds.^[^ ^131^ ^]^ In another study, using BSMV‐mediated sgRNA delivery, *TaHRC* editing provided FHB resistance in wheat without genotype limitation.^[^ ^132^ ^]^ *Fhb1* bioengineering may be applicable to other crops such as maize, which is affected by ear or stalk rot that is reportedly caused by the same fungus.^[^ ^133^ ^]^ *Zm00001d040118* (designated as *ZmFER1*) and *Zm00001d008500*, are two *TaHRC* homologues identified in maize.^[^ ^133^ ^]^ However, analysis of CRISPR/Cas9 mediated mutants of both the orthologues showed that only null mutants of *ZmFER1* exhibited improved tolerance to *Fusarium verticillioides* without compromising yield.^[^ ^134^ ^]^

Bacterial blight, a devastating rice disease, caused by *Xoo*, causes huge yield losses especially in sub‐Saharan Africa and Southeast Asia.^[^ ^135^ ^]^ Rice sugar transporters, *SWEET* genes, that are transcriptionally induced by *Xoo* TALEs, represent major *S* genes to enhance nutrient availability to bacteria and support infection.^[^ ^136^ ^]^ CRISPR/Cas9‐based editing of the susceptible effector binding elements (EBEs) in the promoters of *SWEET* genes: *OsSWEET11*, *OsSWEET13*, and *OsSWEET14*, provided resistance against bacterial blight in rice.^[^ ^137^ ^]^

A highly conserved protein in eukaryotes, translation initiation factor 4E (eIF4E), is manipulated by plant viruses for infection.^[^ ^138^ ^]^ Variant alleles of eIF4E that are unable to interact with host proteins that cause virulence are an important source of recessive resistance against plant viruses.^[^ ^139^ ^]^ A site‐directed‐mutagenized viral‐resistant allele of *eIF4E* when over‐expressed in potatoes provided resistance against *Potato virus Y*.^[^ ^140^ ^]^ Under greenhouse conditions, the homozygous *eIF4E* knockout mutants generated through CRISPR‐Cas resulted in resistance to viral pathogens in cucumbers.^[^ ^141^ ^]^ In tomatoes, the EMS knockout mutants of *eIF4E2* provided resistance against *pepper veinal mottle virus* (PVMV) and CRISPR/Cas9 mutants of *Pelo* conferred resistance against *tomato yellow leaf curl virus* (TYLCV).^[^ ^142^ ^]^ These studies have demonstrated that *S* genes editing can provide effective and durable resistance in plants against viruses that rely on them at some stage of their replication cycle.

*S* gene editing is also emerging as an environmentally friendly way to generate plants resistant to insects/pests. For example, insects rely on plants for specific chemicals such as serotonin, a neurotransmitter from plants that is required for larval immunity and behavior.^[^ ^143^ ^]^ A cytochrome P450 (*CYP71A1*) gene that encodes tryptamine 5‐hydroxylase is involved in tryptamine conversion to serotonin in plants. CRISPR/Cas9 mediated inactivation of *CYP71A1* enhanced resistance to brown planthopper (BPH) by suppressing the synthesis of serotonin and increasing SA levels.^[^ ^143^ ^]^ Similarly, the root‐knot nematodes (RKN) species, *Meloidogyne* graminicola (*M. graminicola* *or Mg*), is one of the most common plant‐parasitic nematodes (PPNs) in rice agrosystems and is responsible for yield losses ranging from 17% to 32% as most rice cultivars are susceptible to it.^[^ ^144^ ^]^ Mutation of rice copper metallochaperone heavy metal‐associated plant protein 04 (OsHPP04) gene with CRISPR/Cas9, resulted in enhanced resistance to the *M. graminicola* without affecting the agronomic performance.^[^ ^145^ ^]^

Since *S* genes are not race‐specific, they are efficient in developing resistance that is effective against multiple pathogens called multiple disease resistance (MDR). MDR can be developed by single *S* gene editing or multiplex editing of *S* genes. An example of developing MDR by single *S* gene editing is Arabidopsis *DOWNY MILDEW RESISTANCE* 6 (*DMR6*), identified in a loss‐of‐susceptibility screen from an EMS population.^[^ ^146^ ^]^ Inactivation of Arabidopsis *DMR6* provided broad‐spectrum resistance to different classes of pathogens, including oomycetes and bacteria.^[^ ^147^ ^]^ Tomato *Sldmr6‐1* mutant plants exhibited increased resistance to fungal, oomycete, and bacterial pathogens.^[^ ^148^ ^]^ Multiplex editing of *S* genes has also been used to develop MDR. For example, CRISPR/Cas9‐mediated generation of the triple mutant of *Pi21, Bsr‐d1*, and *Xa5* resulted in increased resistance to both *M. oryzae* and *Xoo* without affecting agronomic performance.^[^ ^149^ ^]^

#### 3.5.1 Limitations of S Gene Editing and Proposed Solutions

Editing *S* genes is an emerging and efficient strategy for developing resistance in plants in non‐transgenic system. However, engineering several *S* genes simultaneously may be necessary to target a specific pathogen. For instance, multiple genes are involved in FHB resistance, and only partial resistance is provided by *Fhb1*.^[^ ^131^ ^]^ Therefore, in addition to *Fhb1*, other minor resistance QTLs from locally adapted cultivars should be incorporated for higher levels of FHB resistance.^[^ ^211^ ^]^

Another major challenge is the potential developmental problems associated with *S* gene editing, which hinders their application in breeding. Different strategies to finetune their expression and create intermediate alleles, rather than complete loss of function can mitigate this problem.

The gene promoter plays an important role in regulating gene expression by controlling the initiation, termination, and abundance of transcription. Editing promoter provides an excellent strategy to finetune *S* gene expression and avoid developmental abnormalities. For instance, the EBEs in the promoters of the sugar transporter genes from the *SWEET* family were edited using CRISPR/Cas9 technology to prevent pathogen TALEs from binding and activating their expression.^[^ ^137^, ^212^ ^]^ Upstream ORFs (uORFs) are common mRNA elements found in the 5ʹ‐UTR of some genes.^[^ ^213^ ^]^ The uORFs presence is often associated with reduced mRNA translation. In plants, 24%–30% of protein‐coding genes are estimated to contain uORFs.^[^ ^214^ ^]^ The *S* genes can be searched for endogenous uORFs in their 5ʹ‐UTR followed by their modification through genome editing to modulate their expression. Alternatively, de novo uORFs in the 5ʹ‐UTRs have also been used as a strategy to fine‐regulate the plant *lesion mimic mutants (LMMs)* genes.^[^ ^215^ ^]^

Employing genome editing techniques to target multiple sites within a gene can also be used to create optimized variants of the concerned *S* genes. For example, using genome editing techniques, an elite allele of *Lesion mimic mutant (LMM)* gene, *RBL1*, was generated that exhibited broad‐spectrum resistance against both fungal and bacterial pathogens without affecting yield.^[^ ^216^ ^]^ Using CRISPR/Cas9‐based gene editing for single amino acid modification can also potentially improve crops and avoid fitness cost. For example, in rice knocking out of rice *CDPK4* increased resistance to *M. oryzae* but decreased yield. By contrast, enhanced disease resistance as well as increased plant height and improved yield were obtained by editing CDPK4 with a modified amino acid sequence. A similar phenotype was obtained for gene‐edited CDPK18 with altered amino acid sequence.^[^ ^160^ ^]^ The natural recessive mutation, if caused by few bases’ changes, can be copied in susceptible cultivars using genome‐editing technologies such as prime editing and base editing.^[^ ^217^ ^]^ The problems linked to the complete knockout of the *S* genes can also be potentially avoided by generating knockdown alleles using RNAi if the effects on off‐target genes are properly assessed.^[^ ^218^ ^]^ Using structural biology information, *S* genes can be engineered at the specific sites that are potentially involved in pathogenesis.^[^ ^5^, ^97^, ^219^ ^]^

### 3.6 Engineering Plant *Executor* (*E*) Genes for Disease Resistance in Plants

Some phytopathogenic bacteria such as *Xanthomonas* and *Ralstonia* rely on TALE effectors to cause diseases in plants. TALE effectors bind to the specific EBEs in the promoter region of the *S* genes and activate their expressions, causing disease.^[^ ^220^ ^]^ Some plants have evolved a unique type of executor resistance genes, called *executor* (E) genes, to counteract TALEs. The *E* genes, so far, have been reported in rice and pepper only: *Xa7*, *Xa10*, and *Xa23* from rice and *Bs3* and *Bs4c* from pepper. The EBEs in the promoters of *E* genes can specifically trap certain TALEs and trigger strong HR response.^[^ ^18^ ^]^ Broad‐spectrum disease resistance in plants can also be achieved by engineering TALE‐mediated induction of *E* genes.

Many studies have reported the integration of designed EBEs into the promoters of *E* genes to expand the resistance spectrum, or to obtain novel resistance to pathogens in plants.^[^ ^221^ ^]^ However, constitutive expression of *E* genes using conventional transgenic approaches, activated a defense response in the absence of pathogens that caused growth abnormalities. CRISPR/Cas9 homology‐directed repair (HdR) mediated insertion of EBE_AvrXa23_ into the promoter of susceptible *xa23* allele resulted in bacterial blight‐resistance in rice, with normal growth and yield traits.^[^ ^222^ ^]^ Therefore, precisely modifying promoters using gene editing technologies can be exploited for trapping corresponding TALEs to generate disease‐tolerant crops, without growth or yield penalties.

Naturally, a single evolved *E* gene often exhibits race‐specific resistance due to its ability to recognize only one TALE. The evolution of new pathogen variants often overcomes *E* gene‐based resistance. EBE‐stacking‐in‐the‐promoter using genome‐editing technology can be employed for durable and broad‐spectrum resistance and overcome the problem of the evolution of new pathogen variants.^[^ ^223^ ^]^

The precise mechanism of *E* genes inducing HR remains elusive. While, *E* genes have been discovered in few species, it is likely that they are present in all plant species, including the model plant Arabidopsis.^[^ ^224^ ^]^ The discovery of more *E* genes will not only provide a valuable resource for developing disease‐resistant plants but will also help in understanding their mechanism of function and the identification of conserved pattern among *E* genes. Overexpression of *E* genes can develop disease resistance in plants, but since *E* genes are cell death‐inducing genes, the continuous overexpression may pose fitness cost.^[^ ^222^ ^]^ Therefore, strict expression control should be used.

### 3.7 “Silencing” and “Swallowing” Strategy: RNA Silencing and Autophagy for Engineering Disease Resistance in Plants

RNAi in plants acts as a defensive mechanism and a regulatory tool for endogenous genes. While RNAi is not typically effective against prokaryotes due to their lack of RNAi machinery, it has been shown to be effective against fungi and oomycetes.^[^ ^225^ ^]^ Activation of RNAi has proven to be highly effective in the control of plant viruses because they depend completely on plant cellular machinery and are accessible to antiviral plant RNAi. In addition, majority of plant viruses contain single stranded RNA as their genetic material that depend on double stranded RNA (dsRNA) intermediates for their replication. Therefore, transgenic overexpression of viral RNAs in plants leads to the formation of dsRNA which can provide defense against them. This technique called host‐induced gene silencing (HIGS) has been used to engineer virus‐resistant papaya and squash varieties.^[^ ^226^ ^]^ Non‐specific host gene silencing, when using the RNAi for virus resistance in plants can be minimized by using artificial microRNAs (miRNAs).^[^ ^227^ ^]^

While HIGS is effective for controlling plant viruses, difficulty in transformation of many plants and GMO regulations prevent its broad applicability. To address these concerns, spray induced gene silencing (SIGS) is emerging, which involves spraying exogenous dsRNA complementary to viral RNAs to induce plant antiviral RNAi response.^[^ ^228^ ^]^ SIGS has been applied to target over 10 different important plant viruses in different plant species.^[^ ^229^ ^]^ The stability of dsRNAs is a limitation that requires further research to enhance the efficacy of SIGS.

Autophagy (Macroautophagy) is an evolutionary conserved pathway by which dysfunctional organelles and other cytosol components are delivered to lysosomes or vacuoles for degradation and recycling.^[^ ^16^ ^]^ Autophagy not only maintains cellular homeostasis, but also modulates the host response to pathogen infection. It provides an effective defense strategy by targeting viral components for degradation and inhibiting viral replication in plants. The overexpression of autophagy components in plants enhances resistance to viruses while the down regulation of autophagy components suppresses resistance.^[^ ^1^ ^]^ For instance, a virulence factor bC1 from *Cotton leaf curl Multan virus* (CLCuMuV) interacts with autophagy‐related protein 8 (ATG8) and is targeted to degradation. Silencing of autophagy‐related genes *ATG5 and ATG7* reduced plants resistance to viral infection.^[^ ^230^ ^]^ Plant viruses can counteract by hijacking host autophagy by targeting host defense factors for degradation. For example, poleroviruses encoded P0 proteins mediate the degradation of AGO1, a key component in antiviral RNA silencing defense in plants.^[^ ^231^ ^]^

While autophagy plays a key role during plant‐virus interactions, the precise mechanism by which autophagy mediates plant antiviral defense, or how viruses manipulate autophagy for their benefit is largely unknown. Additionally, practical studies aimed at engineering autophagy to obtain disease‐tolerant plants, specifically virus‐resistant plants are needed.

### 3.8 Sustainable and Eco‐Friendly Methods to Control Plant Diseases

While breeding resistance through manipulating genetic pathways offers an excellent strategy to enhance disease resistance in plants, it also increases the risks of the emergence of resistant pathogens and pests. Therefore, alternative strategies that offer more sustainable and eco‐friendly ways to control plant diseases are needed. One such alternative is the use of soil borne, non‐pathogenic rhizospheric or endophytic microbes for the for the management of plant diseases. These beneficial microbes compete with pathogenic microbes through producing toxins and antibiotics and limit their accessibility to nutrients.^[^ ^232^ ^]^ Additionally, exposing plants to beneficial microbes or chemical inducers trigger induced resistance (IR) which can provide broad‐spectrum protection against a variety of pests and diseases.^[^ ^7^, ^233^ ^]^ For instance, non‐pathogenic plant growth‐promoting rhizobacteria (PGPR) and fungi (PGPF) and mycorrhizal fungi are found to trigger IR in plants. The IR phenotype triggered by these microbes is based on the jasmonate (JA) and ethylene (ET) pathways, unlike SAR which depends on SA and expression of *PR* genes^[^ ^234^ ^]^. After their successful application in green house and field trials, interest in using beneficial microbes to control plant diseases is growing. Indeed, many products are commercially available that include spore‐forming bacteria such as bacilli (*Bacillus* spp. And *Paenibacillus* spp. group) or spore‐forming fungi (*Trichoderma, Piriformospora*, and arbuscular mycorrhizal fungi).^[^ ^7^ ^]^ Despite offering an eco‐friendly method to control plant diseases, the resistance provided by beneficial microbes is not complete. However, using them in combination with other genetic methods will reduce the risks of emergence of resistant pathogens and pests.

Beside using beneficial microbes to control plant diseases, plant immunity can be enhanced by using organic plant immunity inducers such as oligosaccharides (that normally function as PAMPs or DAMPs), pathogen‐derived proteins and peptides, unsaturated fatty acids, and various plant defense hormones and functional analogs, including SA and its derivatives, JA and brassinosteroids (BRs).^[^ ^235^ ^]^ Although plant immunity inducers can provide sustainable prevention of plant diseases, their slow effect to induce immunity, lack of studies on field‐scale experiments and challenges in commercial production currently limit their applicability. Further research is needed to overcome these limitations.

Good farming practices such as crop rotation in which members of different families are rotated overtime, reduces disease incidence significantly, because different family members are not equally susceptible to the same pathogen. In addition, inclusion of organic amendments to the soil increases the number and diversity of microbes in plants rhizosphere, which compete with pathogens for nutrients and through antibiosis, helping in plant disease prevention. Moreover, proper sanitation of the fields, and sterilization of soil for preparing nursery bed, can further contribute to combat plant diseases in an eco‐friendly way.^[^ ^236^ ^]^

Biosynthesized nanomaterials (Biogenic NMs) are also promising candidates to achieve agricultural sustainability, food security, and environmental conservation. They have shown effective in controlling a wide range of phytopathogens, including bacterial, fungal and viral pathogens in important crops such as rice, sorghum, soybean, tomato, potato, corn, wheat and legumes.^[^ ^237^ ^]^ However, further research is required to ensure the safety of these nanomaterials, develop scalable production methods and reduce production costs.

### 3.9 Comparative Analysis of Engineering Strategies for Disease Resistance in Plants

All the methods of engineering disease resistance in plants discussed here have pros and cons associated with them. Transferring single resistance gene has been effective but their low recognition range and rapid evolution of pathogens make them less durable. Though gene stacking can overcome the limitations of single‐gene transfer, it may lead to the evolution of “super pathogens” and can result in uncontrollable disease outbreaks. Developing resistance through engineering of immune receptors (PRRs and NLRs) is promising, but it is complex and it is associated with autoimmunity.^[^ ^87^ ^]^ Moreover, the dynamic evolution in effector repertoire of pathogen renders NLRs based resistance less durable.^[^ ^1^ ^]^ Extensive research is still needed before practically using this approach for resistance in field. RNAi has proven effective against viruses but its efficacy against other pathogens such as oomycetes and fungal pathogens is still emerging.^[^ ^1^ ^]^ Engineering autophagy provides a powerful tool against pathogens particularly viruses but the mechanism is not properly understood yet and practical studies at exploiting autophagy to obtain disease tolerant plants are still lacking.^[^ ^16^ ^]^ Most importantly, all the above methods involve resistance generation in transgenic system or involve extensive genetic changes, which is making them less popular among general public.

Compared to other methods, *S* gene‐editing is providing the best genetic approach for engineering disease resistance in plants. *S* gene‐editing provides resistance in non‐transgenic system which is increasingly accepted by the public. In several countries, including United States, gene‐edited crops that do not contain foreign DNA are not regulated as GMOs.^[^ ^238^ ^]^ It is hoped that other countries will also adopt them soon. Additionally, the resistance obtained by *S* gene‐editing is long‐lasting and to overcome *S* gene‐based immunity, pathogens have to evolve a completely new function that can replace the *S* gene. Eco‐friendly sustainable strategies ‐ are getting popularity but they are not very effective.^[^ ^7^ ^]^ However, using them in combination with genetic approaches can help mitigate the emergence of resistant pathogen populations.

## 4 Conclusion and Perspectives

**Figure 5:**
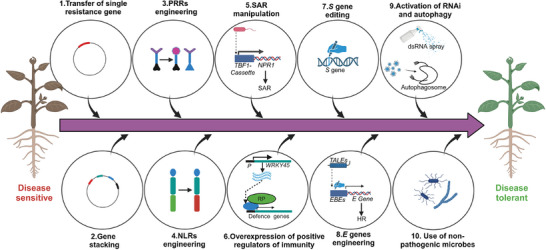
Pathways to disease resistance engineering in plants. Transfer of single resistance (Using traditional approaches or modern transgenic approaches) is used to develop disease resistance in plants 1). Gene stacking is used to cope with the pathogens that have overcome the resistance due to single gene transfer 2). Domain swapping in PRRs is another strategy to develop disease resistance in plants 3). NLR engineering is a popular strategy to enhance disease recognition or develop new‐to‐nature disease resistance in plants 4). Components of SAR such as *NPR1* or *PR* genes can be over‐expressed under stringent control promoters to develop disease resistance 5). Overexpression of specific positive regulators of defense response such as TFs is also a potential strategy to engineer resistance if pleiotropic effects can be avoided 6). Pathogens manipulate host genes called *S* genes to their benefit, causing infection. The *S* gene editing in order to stop “helping pathogens” is thus a popular strategy to develop disease resistance in plants 7). Plants have evolved *E* genes to trap certain transcription activator‐like effectors (TALEs). The effector binding elements (EBEs) of *E* genes can be engineered to enable them to trap TALEs or other effectors thus enhancing disease resistance in plants 8). Activation of RNAi and autophagy can provide powerful strategies to control plant diseases particularly those caused by plant viruses 9). The use of beneficial microbes and organic methods are providing eco‐friendly and sustainable strategies for plant disease management 10).

Excessive crop losses due to diseases and the emergence of resistant pathogen strains necessitate a deeper understanding of plant‐pathogen interactions and the development of alternative pathogen management methods. Developing immunity through the transfer or engineering of immune receptors is beneficial as they are directly targeted by pathogens and pests. However, resistance provided by the transfer of a single gene coding for resistance can be rapidly overcome due to pathogen evolution. Gene stacking or pyramiding can address this issue. Domain swapping in PRRs or engineering NLRs using strategies such as decoy engineering, integrated domain engineering or proteolytically activated synthetic NLRs, can enhance recognition specificities and even create new‐to‐nature resistance in plants. Another emerging strategy is *S* gene editing, especially if fitness cost can be avoided. Additionally, manipulating SAR components such as NPR1 or NHP biosynthesis could be employed to engineer broad‐spectrum plant resistance. Activating RNAi and autophagy pathway can provide a powerful tool to control phytopathogens particularly viruses. Moreover, there is growing interest to control plant diseases using sustainable and eco‐friendly ways, such as by using beneficial microbes, organic immunity inducers, biogenic nanomaterials as well good farming practices (**Figure** **5**).

There is a need to screen for more resistant resources to achieve the desired resistance. Wild relatives serve as an excellent alternative if resistance is not available in current cultivars.^[^ ^32^ ^]^ Studies aimed at enhancing disease resistance or generating novel resistances using decoy engineering, integrated domain engineering/replacement, or proteolytically activated synthetic NLRs have mostly been performed in model plants, and need to be carried out in established crops such as wheat, rice, maize, and barley. Additionally, investigating the structural basis of plant‐pathogen interactions and the mechanisms of downstream signaling is necessary to overcome autoimmunity in the case of immune receptor engineering or developmental defects in the case of *S* gene editing.

With the expanding understanding of PAMPs, effectors, PRRs, and NLRs, facilitated by AI advancements, we anticipate the potential to predict interactions among PAMPs and PRRs, as well as effectors and NLRs. This progress may lead to the realization of “synthetic plant disease resistance,” enabling the *in‐silico* synthesis of PRRs, NLRs, and even *S* genes. Additionally, rather than targeting individual evolving effector, targeting the effector translocation machinery with the help of genome editing aided by AI technologies can be exploited to develop broad‐spectrum and durable disease resistance.^[^ ^239^ ^]^ The recently released database of disease susceptibility genes in plants (DSP), can assist the researchers by providing an advanced and simple search tool for the identification of *S* genes in plants.^[^ ^240^ ^]^ Recently, biomolecular condensates, which are dynamic nonmembranous structures, have emerged as important players in plant‐pathogen interactions.^[^ ^241^ ^]^ Targeted regulation of these condensates will create more opportunities to improve plant disease tolerance.^[^ ^242^ ^]^ All these strategies could empower plants to recognize various pathogens or prevent pathogen assistance, ultimately bestowing disease resistance.

## Conflict of Interest

The authors declare no conflict of interest.

## Author Contributions

A.U.I. and M.S.S.K. contributed equally to this work. C.J. and A.U.I. conceived the study and designed the manuscript structure. A.U.I. prepared the figures. A.U.I., M.S.S.K., F.I., S.A., T.L., and F.F. wrote the manuscript. Z.S., H.C. and C.J. revised the manuscript. All authors contributed substantially to this article's writing and agreed with the submitted version.
